# Deuterium‐Oxide Labelling Proteomics Illustrates the Importance of Sample Timing and the Limitations of Serial Biopsies

**DOI:** 10.1002/jcsm.70426

**Published:** 2026-11-10

**Authors:** Colleen L. O'Reilly, Michael E. Taylor, Kamil A. Kobak, Ying Ann Chiao, Rovshan G. Sadygov, Michael T. Kinter, Benjamin F. Miller

**Affiliations:** ^1^ Aging and Metabolism Research Program Oklahoma Medical Research Foundation Oklahoma City Oklahoma USA; ^2^ Department of Biochemistry and Physiology University of Oklahoma Health Campus Oklahoma City Oklahoma USA; ^3^ Department of Biochemistry and Molecular Biology The University of Texas Medical Branch Galveston Texas USA; ^4^ Veterans Affairs Medical Center Oklahoma City Oklahoma USA

**Keywords:** biopsy, deuterium oxide, proteomics, skeletal muscle, stable isotopes

## Abstract

**Background:**

Measuring in vivo protein synthesis is important for testing how interventions, disease and ageing remodel the proteome. A limitation of approaches using short‐term infusions of labelled amino acids was that long‐term changes had to be extrapolated from short‐term measurements. Deuterium oxide (D_2_O) is now widely used because it can measure protein synthesis over periods of days to weeks. We previously showed that the duration of labelling impacts measured protein synthesis, but many still employ repeated tissue sampling during D_2_O provision. We hypothesized that the timing of tissue sampling alters which proteins contribute to the overall protein fractional synthesis rate (FSR), thereby invalidating comparisons between serial samples collected during continuous labelling.

**Methods:**

To test this hypothesis, we used a five‐timepoint (0d, 4d, 15d, 30d and 45d) D_2_O labelling time course followed by liquid chromatography–tandem mass spectrometry proteomics to calculate individual protein synthesis rates and plateau values in mouse gastrocnemius muscle. Individual proteins were stratified into slow and fast groups, such that each group was responsible for the slowest or fastest 20% of total synthesis over the first 4 days of labelling, respectively.

**Results:**

In a 4‐day period representing a baseline period, the synthesis rate of summed proteins was 2.09%/day. When the period was extended to 15, 30 or 45 days, the synthesis rates of summed proteins declined to 1.64%, 1.31% and 1.10%/day, respectively. The contribution of slow and fast proteins to these rates changed as a function of time. The fast and slow proteins accounted for 4.3% and 34.0% of measured proteome abundance, respectively. From Day 0 to Day 4, fast and slow proteins contributed 19.8% and 20.2% of the calculated FSR, respectively. By Day 45, these contributions shifted to 6.5% and 28.8%. Further, we demonstrated that the assumption that the fraction new of proteins linearly increases to 100% new is not true for the majority of proteins. Instead, most proteins plateaued at some point less than 100%, which stops their individual contribution to a summed protein synthesis rate.

**Conclusions:**

In continuous long‐term labelling studies, biopsy timing changes the measured bulk FSR because the relative contributions of individual proteins to that measurement over time. Fast proteins that plateau early decrease their contribution as labelling time increases. These findings indicate that studies with different labelling periods, or multiple biopsies over time during continuous labelling of days to weeks cannot be compared to biopsies taken at other times in the labelling period.

## 1 Introduction

In recent decades, considerable attention has been devoted to methods for measuring muscle protein synthesis in humans [1, 2, 3, 4, 5, 6, 7]. Early investigations favoured the measurement of protein synthesis using the infusion of isotopically labelled essential amino acids (^13^C or ^2^H leucine or phenylalanine) over short periods (hours) followed by tissue sampling [8, 9]. Amino acid tracers were an important step in understanding protein synthesis but required an inference of long‐term changes from short‐term outcomes [9, 10, 11, 12, 13]. Since these initial studies, deuterium oxide (D_2_O) became a widely used approach due to its potential for use in both short‐term experiments and free‐living measures in humans over days [14, 15] to weeks [16, 17]. This ability for use in both short‐ and long‐term labelling paradigms makes D_2_O a powerful tool for studying protein synthesis in complex and heterogeneous tissues with broad synthesis rates, such as skeletal muscle.

The principles of the D_2_O approach are similar to those of traditional labelled amino acid infusion methods, in that the fractional synthesis rate can be measured by labelling a precursor molecule pool that is incorporated into a product molecule over time [9]. In comparison to labelled amino acids, determination and calculation of the precursor pool is simplified by the equilibration of D_2_O with the body water pool and amino acids, which reach a steady‐state precursor pool enrichment rapidly after labelling begins [2]. Therefore, it is common for many in the field to apply the traditional amino acid labelling experimental design to extended labelling periods and repeated biopsies to examine responses to conditions over time [18, 19].

As we demonstrated previously, the length of the labelling period determines the contribution of individual proteins to an integrated synthesis rate [9, 20]. When we modelled outcomes from different labelling periods, we showed that a short labelling period of 20 min resulted in 29% higher rates of synthesis compared to labelling over 6 weeks [20]. The differences in synthesis rates between labelling periods is largely because a single measurement of ‘protein synthesis’ is the average synthesis rate of thousands of individual proteins, each with different abundances and synthesis rates. Many in the muscle field, including us, use tissue fractionation methods (e.g., myofibrillar, mitochondrial and cytosolic) to have more uniform protein pools in hopes of minimizing heterogeneity. However, the assessment of these pools being faster (usually ascribed to mitochondria) or slower (usually ascribed to myofibrillar) are just based on the average rate measured for those fractions without distinction of the individual rates of the proteins within the fraction. In reality, those fractions also contain both fast and slowly synthesized proteins.

In proteins that fully renew, the isotope tracer is incorporated until it reaches the enrichment of the precursor pool. When the protein reaches the precursor enrichment it is fully labelled, or saturated. Saturated proteins have not stopped turning over; rather, their enrichment does not increase further because it has reached the maximal labelling. Proteins with fast synthesis rates accumulate label rapidly, thus reaching label saturation quickly and no longer increase the measured fraction new of the total protein pool. Therefore, fast proteins have an outsized contribution to bulk label accumulation at short time periods. Some suggest that unless the measured time is less than 24 h, fast‐turning‐over proteins will not impact bulk synthesis rates [18, 19]; however, this suggestion has not been experimentally tested.

In addition to the differences in synthesis rate, there is also a need to consider the fraction of a protein pool that renews. Standard fractional synthesis rate (FSR) calculations use a single timepoint during the period of labelling with the assumption that the timepoint is on the linear phase of label incorporation and that the product enrichment will eventually equate to the precursor enrichment (i.e., reach 100% new). We developed a time‐course labelling approach to determine when and if proteins fully renew [21]. This approach determines at what fraction new a given protein pool reaches a plateau. The value at which that plateau occurs is the fraction of the pool that renews, and reciprocally what fraction of the pool is not renewing. In our first study, we showed that in one muscle (gastrocnemius), only 14%–26% of collagen renewed [21]. Additionally, we demonstrated that myofibrillar proteins got close to, but did not reach, 100% renewal. More importantly, we showed that not accounting for this plateau value when calculating synthesis rates could change the findings of a study [21]. We recently confirmed our previous findings in an experiment where old rats underwent hindlimb suspension and found that the dynamic protein pool of collagen was smaller in the old muscle than in adult muscle [22]. We again found that neither myofibrillar nor collagen proteins fully renewed, with the incomplete renewal being higher in collagen [22]. Others have shown this in mouse liver as well [23]. Therefore, proteins that plateau at a different fraction of the protein pool, or reach their plateau at a different timepoint, could influence bulk measurements of synthesis.

Some studies take advantage of the long‐term nature of D_2_O labelling to compare control and intervention periods by taking biopsies at two different points [18, 24, 25]. In these studies, the time since the previous biopsy is treated as the labelling interval used to calculate FSR for that labelling period. In some cases, the labelling period is the same, but other times, it is different [18, 24, 25]. Since we have previously shown that the period of labelling influences bulk synthesis rates [20], such a comparison seems invalid. Further, these study designs assume that synthesis occurring between serial biopsies linear, but the two biopsies are at different points along the saturation curve for specific individual proteins or groups of proteins. This concept becomes particularly important when subtracting out enrichment that is already present from the ‘pre’ sample because enrichment of the tissue will also depend heavily on the heterogeneity of protein synthesis rates of individual proteins within the sample at the ‘pre’ timepoint versus the post. Despite prior discussion of the importance of label duration [9, 24, 26], no study has examined how serial biopsy timing impacts bulk synthesis rates. To test this potential impact, we hypothesized that the timing of tissue sampling alters which proteins contribute to mixed protein FSR, thereby invalidating direct comparisons between serial samples collected during continuous labelling.

## 2 Methods

Methods are provided in brief with full details available in the Methods section in the Supporting Information.

### 2.1 Animals

All animal procedures were conducted in accordance with institutional guidelines for the care and use of laboratory animals and were approved by the Institutional Animal Care and Use Committee at the Oklahoma Medical Research Foundation (OMRF). To avoid the logistical and practical constraints associated with multiple biopsies, we used control mouse muscle [27] to test our hypothesis. Old (23‐month) male C57Bl/6 mice were housed in the vivarium at OMRF. Animals were group housed (a maximum of five per cage) with ad libitum access to food and water in a room on 14:10 light:dark cycle with constant temperature and humidity. Prior to sacrifice, mice were subjected to 4‐h fasting. For tissue and blood collection, mice were anaesthetised with isoflurane followed by exsanguination through cardiac puncture for euthanasia.

### 2.2 Experimental Design

To compare how the period of labelling impacts the contribution of individual proteins to an integrated protein synthesis rate, we used labelling periods of 4, 15, 30 and 45 days (*n* = 3 mice/labelling period). At the onset of the labelling period, mice were given an intraperitoneal bolus injection of filtered 99% D_2_O (Sigma Aldrich. St. Louis, MO) equivalent to 5% of body water, estimated at 60% of body mass. The bolus injection was followed by 8% D_2_O‐enriched drinking water for the remainder of the labelling period. At the end of the labelling period animals were euthanized when blood from a cardiac puncture was collected into EDTA‐lined tubes. Blood was centrifuged at 3000 × *g* for 5 min at room temperature. Serum was aliquoted and frozen at −80°C until further analysis. The gastrocnemius was subsequently removed, weighed, flash frozen and stored at −80°C (Figure 1).

**FIGURE 1:**
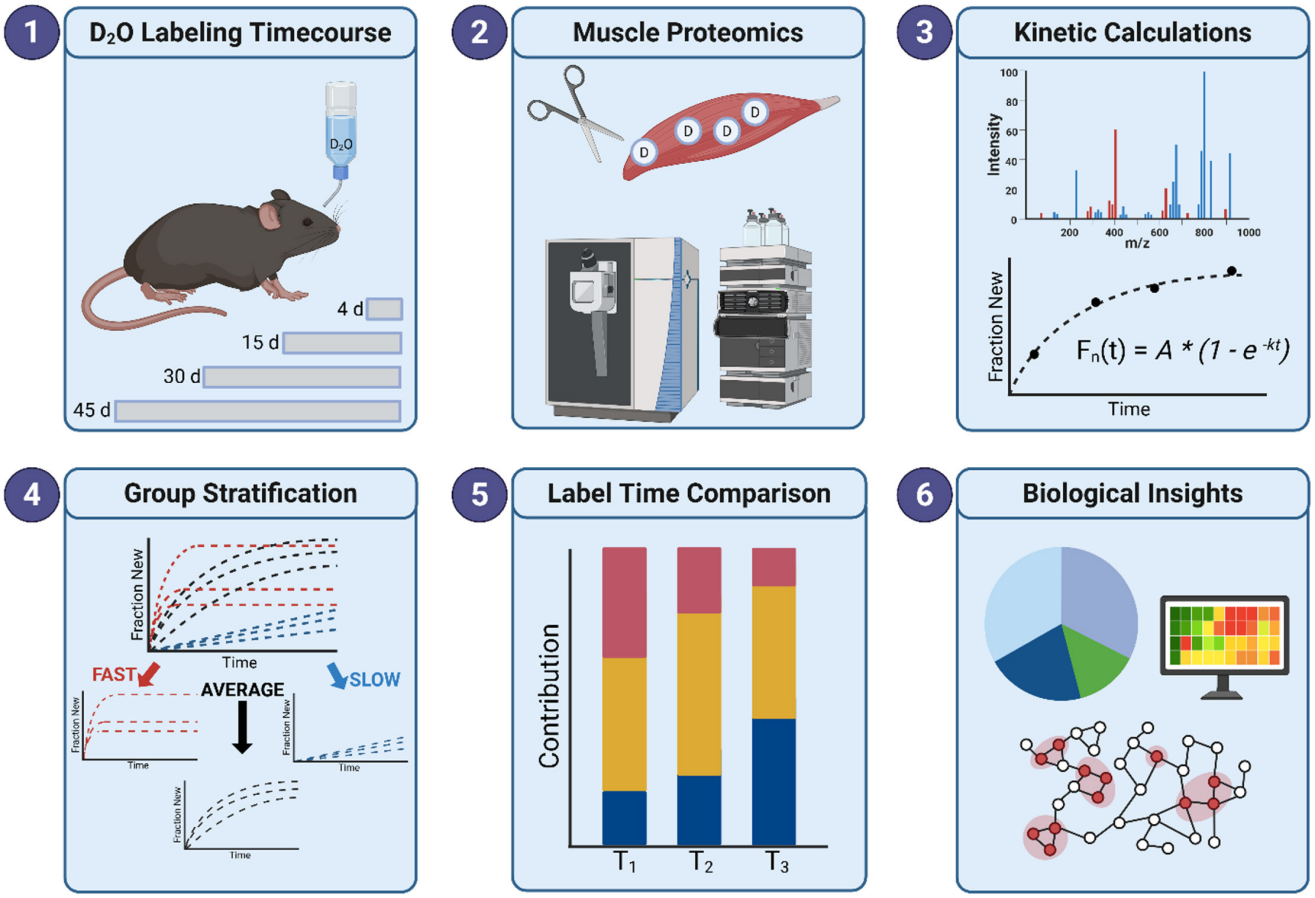
Experimental design and data processing workflow. Mice were provided with deuterium‐enriched water, leading to deuterium incorporation into newly synthesized proteins. Gastrocnemius muscles were collected and analysed by LC–MS/MS proteomics. From the mass spectrometry data, deuterium incorporation was quantified for each detected protein to estimate the protein synthesis rate constant (*k*) and labelling asymptote (*A*). Proteins were then classified into ‘fast’, ‘average’ and ‘slow’ kinetic groups. The contribution of each group to the overall synthesis signal was evaluated across multiple labelling windows, and the implications for biological interpretation were assessed.

### 2.3 Body Water Enrichment

We determined body water enrichment as previously described [28].

### 2.4 Sample Processing

Frozen powdered skeletal muscle (~30 mg) was lysed in 1‐mL RIPA buffer and sonicated for 15 cycles of 30 s on/off (Bioruptor Plus; Diagenode, Denville, NJ). Samples were incubated at 90°C and 800 rpm for 10 min, and then the sonication was repeated. Then samples were centrifuged for 5 min at 500 × *g* to remove debris. The supernatant was collected, and the protein concentration in sample was measured using Pierce 660 Protein Assay. Eighty micrograms of total protein was taken for analysis, and 1 μg of BSA was added to the protein samples as an internal standard. LC–MS/MS data acquisition details are included in the Supporting Information.

### 2.5 LC–MS/MS Data Analysis

LC–MS/MS files were analysed using DIA‐NN 2.2.0 [29, 30]. Following DIA‐NN analysis, the main report was filtered for peak quality and retention time (specifics are supplied in the Supporting Information). The filtered main report was then imported into Skyline, where peaks were integrated and peak areas were exported. Analysis was completed on 2826 peptides across 308 proteins.

### 2.6 Protein Synthesis Rate Calculation

The calculation of protein synthesis rates and asymptotes is shown in full in the Supporting Information. In brief, we used mass shift caused by the deuterium labelling of each peptide to calculate the fraction new of each peptide. Once the fraction new of each peptide was determined at each timepoint, the protein fraction new value was calculated as the mean of the peptide fraction new values. Peptides were filtered for quality prior to being included in the mean (see Supporting Information). Then, by fitting the following equation to the fraction new value of a protein at each timepoint, *k* (rate) and *A* (asymptote) values were determined:

$$\text{Fraction}\;\mathrm{New}=A*\left(1-e^{-\mathrm{kt}}\right)$$

### 2.7 Bulk and Myofibrillar FSR Estimation

To re‐capitulate the myofibrillar and total‐muscle (i.e., bulk) FSR measurements commonly reported from GC–MS analyses of hydrolysed whole muscle or myofibrillar fraction, we determined the fraction new of each protein, or proteins represented in a myofibrillar fraction sample (Supplementary Table S1), at each timepoint and created an abundance‐weighted sum of these values to calculate a proteome‐wide fraction new at each timepoint. The FSR at each timepoint was calculated by dividing the fraction new by the length of the labelling period.

### 2.8 Gene Ontology Enrichment Analysis

After LC–MS/MS analysis all proteins found to meet all quality control requirements were included in our Gene Ontology analysis (GO). The list of proteins was analysed using GO enrichment analysis for cellular component. Proteins could appear in multiple categories. To determine the cellular component makeup of the proteome, as defined by GO, we calculated the abundance‐weighted protein hits per category. Each protein contributes its full abundance to each category it belongs to but is only counted once within a category. This approach allows the contribution of each protein to reflect its relative abundance in the proteome while still permitting proteins with multiple annotations to contribute to all relevant GO categories. We determined this would provide the most physiologically representative view of proteome composition.

To account for the influence of protein synthesis kinetics on functional representation at a given sampling time, we quantified GO category composition separately within each synthesis group (fast, average, slow). For each group, we calculated the fraction of total protein abundance attributable to each GO category. This approach allowed us to determine how functional representation differs as a function of synthesis speed, thereby illustrating how proteins contributing to GO categories would vary across sampling intervals.

### 2.9 Generative AI Usage

Generative AI was used to assist in the creation of the R scripts used for this manuscript.

## 3 Results

To demonstrate how labelling duration impacts measured protein synthesis rates, we mimicked a typical study design. First, the FSR (%/day) was calculated between Day 0 and Day 4 to be indicative of a pre‐ or baseline period. The proteome‐wide FSR from Day 0 to 4 was calculated by dividing the Day 4 fraction new by 4, giving an FSR of 2.09%/day. FSR was then calculated over the full labelling windows from Day 0–15, Day 0–30 and Day 0–45, as well as over the corresponding second windows from Day 4–15, Day 4–30 and Day 4–45. Second‐window FSRs were calculated by subtracting the Day 4 fraction new from the fraction new measured at each later time point and dividing by the duration of the second interval. The Day 0–15 and Day 4–15 FSRs were 1.64%/day and 1.48%/day, respectively. The Day 0–30 and Day 4–30 FSRs were 1.31%/day and 1.19%/day, respectively. The Day 0–45 and Day 4–45 FSRs were 1.10%/day and 1.00%/day, respectively (Figure 2). We further performed analyses between the different timepoints to illustrate how the different labelling periods affect apparent synthesis rates for both the bulk fraction and myofibrillar fraction (Supplementary Figure S1). Since both the bulk fraction and the myofibrillar fraction were similarly influenced by time, we only present the bulk fraction for the remainder of the data. The bulk synthesis rate shown in Figure 2 represents a cumulative average synthesis rate across hundreds of different proteins with heterogeneous synthesis rates. To demonstrate the heterogeneity of the ‘bulk’ rates we stratified individual proteins into three synthesis groups—slow, average and fast. Proteins were rank‐ordered by rate constant (*k*), and contribution to total fraction new at Day 4 was computed as the product of its fractional abundance and its fraction new. Summing these abundance‐weighted contributions across all proteins gives the total fraction new of the measured proteome. Proteins were then partitioned along this *k*‐ranked axis with the goal of stratifying slow/average/fast proteins into groups. Our target distribution of proteins was 20/60/20 (slow/average/fast); however, the final proportions differed slightly because the proteins at each boundary were not divided between groups. We therefore ended up with 20.2% of total fraction new designated the slow group, 19.8% of the fastest turning‐over proteins designated the fast group and the remaining 60% of fraction new designated the average group. This approach yielded 30, 190 and 88 proteins in the slow, average and fast groups, respectively. Synthesis group characteristics are described further in Supplementary Figures [Link], [Link], [Link]. The contribution of the fast proteins (red) and the average proteins (yellow) to the measured FSR was highest at shorter labelling periods and decreased with longer labelling durations (Figure 3A). In contrast, the contribution of slow proteins (blue) increased modestly with the labelling period to the observed FSR (Figure 3A,B). To visualize these differences in relative contribution, we then measured the percent contribution of each group of proteins (fast, slow and average) to the ‘bulk’ FSR at each time point (Figure 3B). At Day 4, the fast proteins were responsible for 19.8% of the calculated FSR but only contributed 6.5% when labelling was extended to 45 days. In contrast, the slow proteins were responsible for 20.2% of the FSR at Day 4 and increased to 28.8% when labelling was extended to 45 days. The average proteins were responsible for 60.0% of the FSR at Day 4 and 64.7% when labelling was extended to 45 days. Taken together, these data indicate that increased labelling duration not only lowers the ‘bulk’ FSR but also alters the composition of the proteins that contribute to the measured FSR.

**FIGURE 2:**
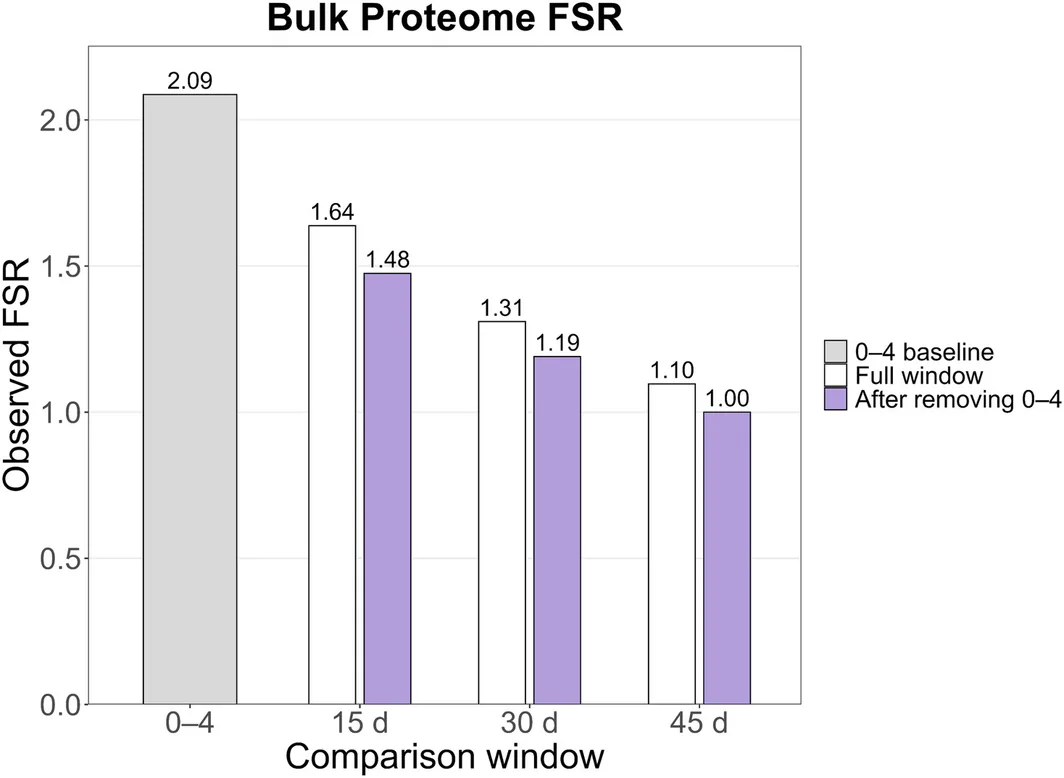
The effect of the labelling time period on observed FSR. The grey bar represents the FSR observed from Day 0 to Day 4, calculated by dividing the proteome fraction new at Day 4 by four. The white bars show the FSR that would be calculated over a series of longer labelling periods, while the purple bars show the FSR that would be calculated if the labelling from Day 0 to Day 4 was subtracted from the labelling observed at the end of the labelling period, as would be done in a serial pre–post study design.

**FIGURE 3:**
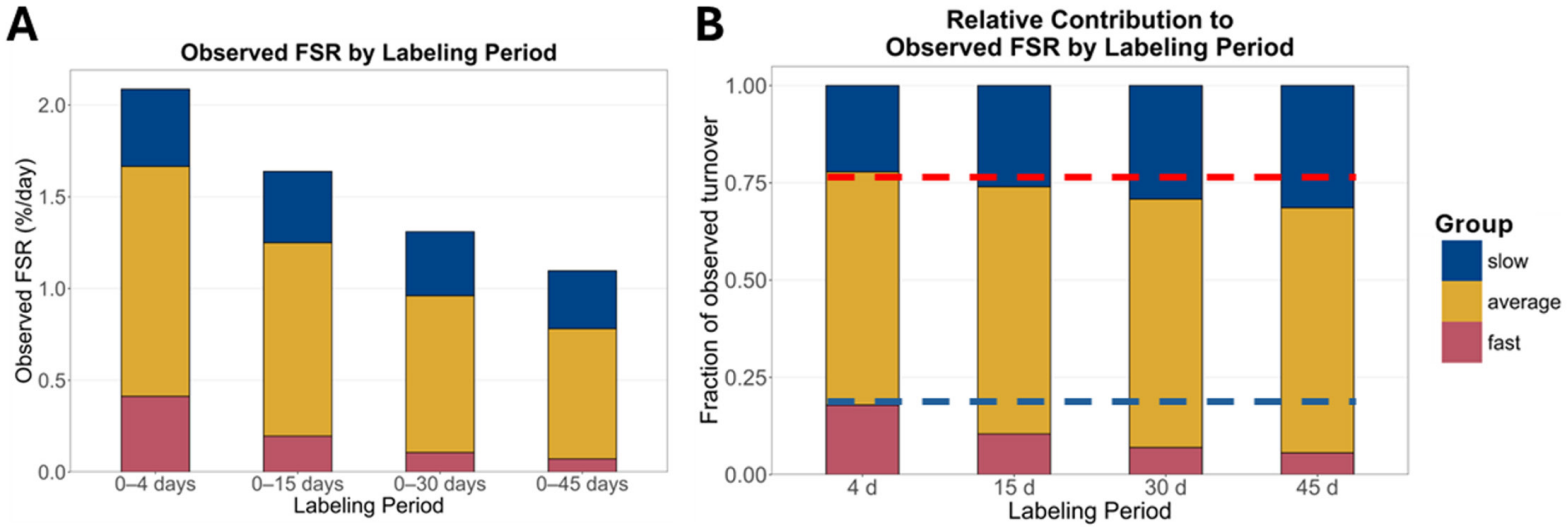
Labelling period changes the relative contribution of proteins to ‘bulk’ fraction new. (A) Measured FSR stacked by kinetic group for cumulative labelling windows. (B) The relative contribution of each synthesis group at each labelling timepoint. Dashed lines indicate the values at 4 days of labelling.

Next, we looked at the fraction new for each protein over the labelling time course. To visualize heterogeneity in individual protein synthesis rates and the renewal plateaus, we plotted the fraction new trajectory of each individual protein over our labelling period (Figure 4A). This representation demonstrates the heterogeneity of the synthesis rates (*k*) and labelling plateau values of individual proteins. The dark line represents the ‘bulk’ or average fraction new of all proteins at each time point while each grey line represents an individual protein. As discussed earlier, we again observed that many proteins did not reach complete synthesis and that the time required to reach plateau varied widely. Next, we looked at the fraction new for each protein over the time course using a heatmap (Figure 4B). The proteins were ordered by synthesis rate and colour coded by synthesis group, with darker colour within each group indicating a higher fraction new. The visualization in Figure 4B highlights the temporal differences in fraction new between both individual proteins and our designated synthesis groups. The fast synthesis proteins reached their darkest colour quickly, while the average and slow proteins did so much more gradually. To further visualize the heterogeneity in both plateau value and time to plateau we separated proteins into their protein synthesis groups as described in Figure 3 and plotted individual traces of fraction new for the proteins within each group (Figure 4C). Within the slow‐synthesis group, a clear plateau is absent, whereas fast‐synthesis proteins show a rapid rise followed by plateau. Considerable variability in fraction new at plateau was observed within all three synthesis groups.

**FIGURE 4:**
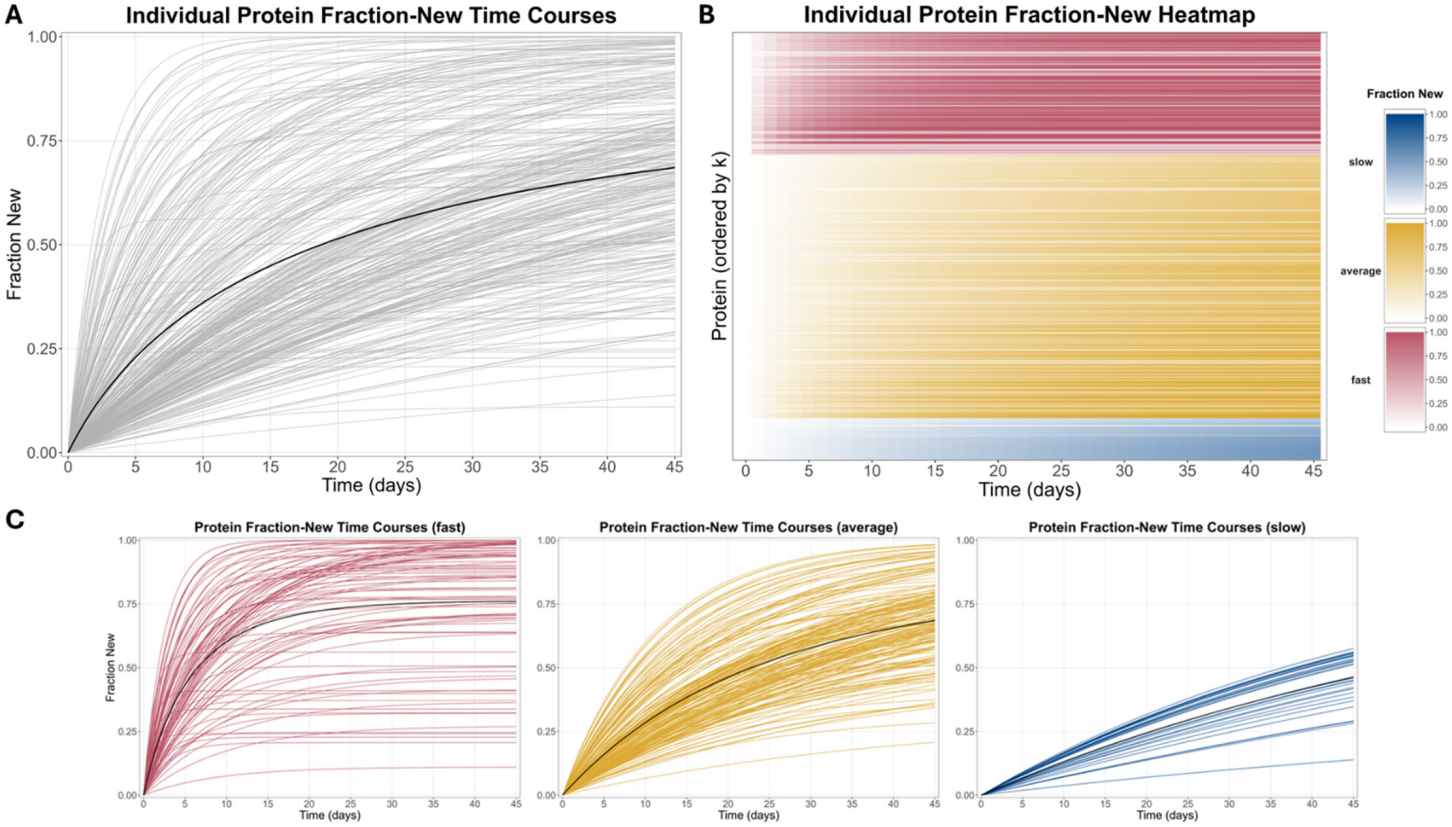
The heterogeneity of muscle proteome synthesis, stratified by synthesis rate. (A) The fraction new traces of each individual protein over the labelling period. The black line indicates the average synthesis rate and plateau value. We observed heterogeneity in both the synthesis rate and the plateau value. (B) The fraction new of each protein over the labelling period shown as a heatmap. Each row represents a single protein, with the colour of the row designating the synthesis group. Protein rows get darker as the fraction new increases. (C) The fraction new traces of each synthesis group—fast, average and slow. The ‘fast’ proteins quickly rise to plateau, while the slow proteins are still approximately linear at the end of the labelling period. The black line indicates the average trace for the group.

To illustrate how the relative contribution of each synthesis group to the measured synthesis shifts across labelling windows, we calculated the fraction of the proteins in each synthesis group that had not yet reached 50% of maximum labelling (Figure 5A). All fast proteins reached 50% synthesis by approximately 7 days, while the slow proteins started to reach 50% labelling at around 35 days. We next examined how the contributions of individual proteins to measured synthesis vary across the labelling period, reflecting what would be observed with serial biopsies over time. For each protein, we computed the derivative of its fraction new curve and expressed it as a percentage of the summed derivatives across all measured proteins at each time point (Figure 5B). The proteins driving further changes in fraction new changed dramatically over the labelling period. To emphasize this, we divided proteins into 10 groups of equal fraction new over the 0–15 day labelling period (Figure 5B, inset) and then calculated how much additional synthesis each group contributed during the 15–45 day period. The contribution of the slowest deciles increased dramatically, while the contribution of the fastest deciles effectively vanished. These measurements are not weighted by relative abundance, as the goal is to highlight the synthesis of individual proteins within the muscle proteome.

**FIGURE 5:**
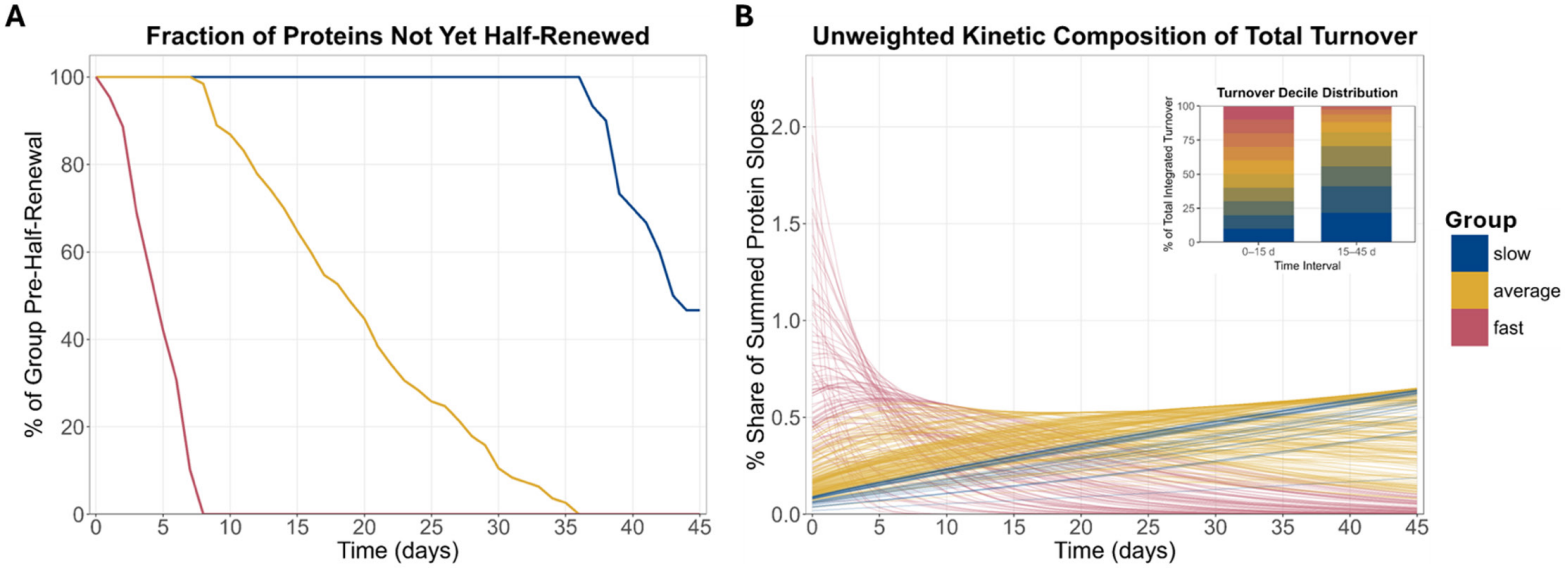
Measured label accumulation is initially dominated by fast proteins, while slower proteins drive further labelling accumulation. (A) The fraction of proteins in each synthesis group that are not yet 50% renewed. Note the rapid dropout of the fast proteins. (B) The unweighted kinetic composition of label accumulation throughout the labelling period, computed by normalizing each protein's labelling rate to the summed rate across all proteins at each time point (values sum to 100% at every time). Inset: Proteins were binned into deciles based on their integrated synthesis signal from 0 to 15 days. Each colour block represents 10% of total label accumulation from 0 to 15 days. The total labelling contribution of the same protein deciles was then calculated from 15 to 45 days, showing how their share of the integrated synthesis signal shifts across time intervals. The area of each decile represents its contribution to total label accumulation during the designated labelling period.

In addition to examining the heterogeneity of rate of synthesis, we also wanted to investigate if the cellular component identification of the proteins was also different among the synthesis stratification. We performed GO analysis of the cellular component of each individual protein, with more than one cellular component being allowed for each protein. The cellular components were then condensed into seven categories (details shown in Supplementary Materials): cytoskeleton/structural, extracellular matrix/junctions, membranes and transport, mitochondrial and oxidative metabolism, myofibrillar/contractile, nuclear and sarcoplasmic reticulum/calcium. The measured abundance of each protein was added to each applicable cellular component category (Figure 6A). The abundance weighted visualization allowed us to examine the different contributions of cellular component categories to our different synthesis rate groups as well as what influence the different contributions across synthesis rates would have within the proteome. Extracellular matrix/junctions contributed the least by abundance to the overall proteome (3.2%), and myofibrillar/contractile contributed the most (29.1%), closely followed by membranes and transport (17.1%) and cytoskeletal (14.8%). However, protein abundance alone does not account for the synthesis rate of each protein and therefore the potential for different representation depending on length of labelling time. Therefore, we calculated what fraction of each synthesis group (by abundance) was attributable to each of the seven cellular components (Figure 6B). For example, greater than 40% of the slow and average group proteins were attributed to myofibrillar/contractile proteins while a much lower percentage (13.4%) of the fast group proteins were identified as myofibrillar/contractile proteins. Our cellular component analysis of each synthesis group shows the diverse contributions of components and function across synthesis speed (slow, average, fast) and visually demonstrates how different time points could result in a different protein population.

**FIGURE 6:**
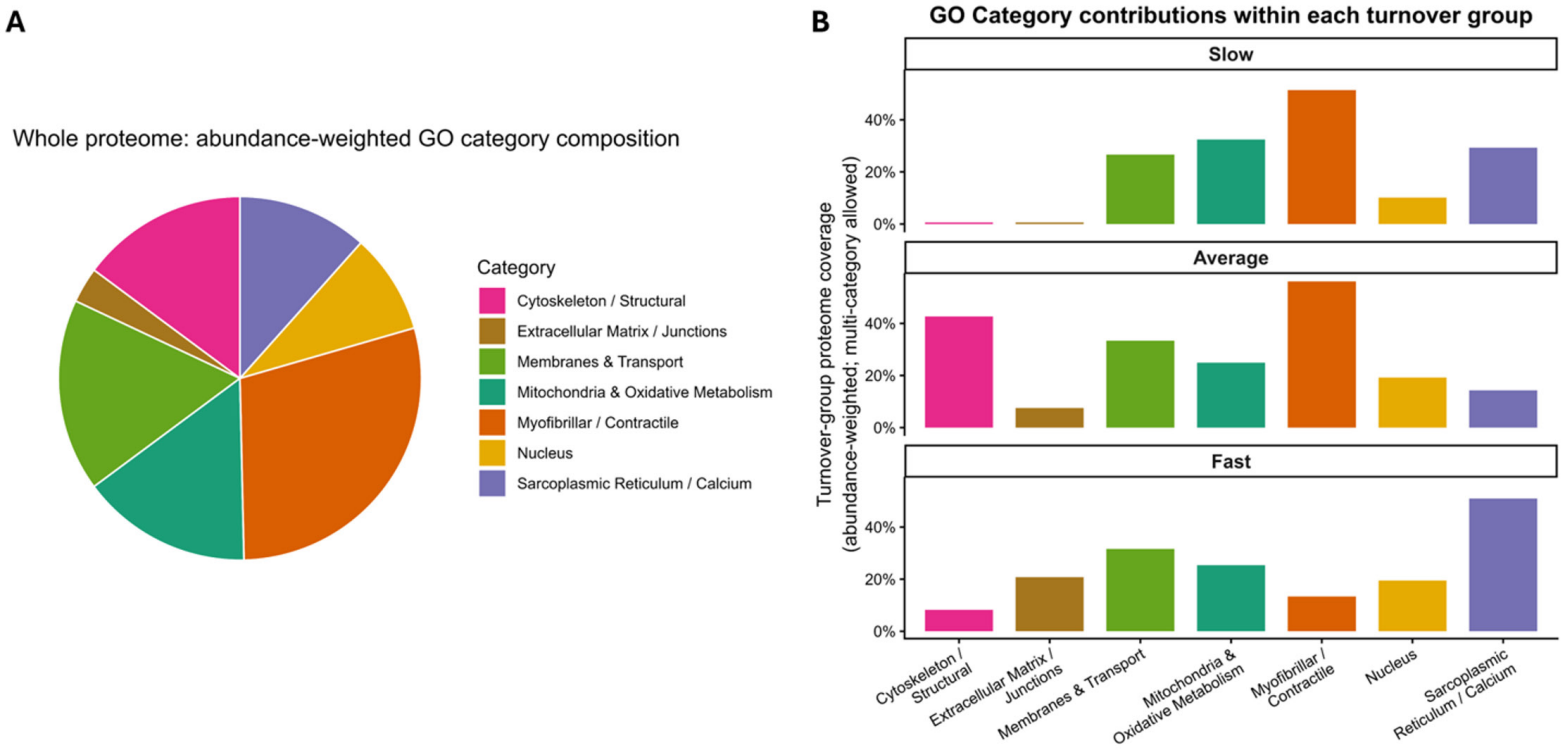
Heterogeneity of the cellular component/function of the proteome and by synthesis rate. (A) Gene Ontology (cellular component) enrichment analysis of the detected proteome, weighted by protein abundance. Proteins (and their abundances) may contribute to multiple GO categories. Pie‐slice areas are proportional to each category's abundance‐weighted contribution, computed as the product of the total GO membership count and the summed abundances of proteins assigned to that category. (B) The fraction of total abundance within each synthesis group that contributes to each cellular component category.

## 4 Discussion

Measuring bulk and myofibrillar protein synthesis in skeletal muscle is important for answering questions related to the gain, preservation and loss of muscle mass. Evaluation of therapeutic interventions relies on valid measurements of protein remodelling. In this study, we demonstrated that duration of labelling period influences the measured rate of bulk and myofibrillar FSR and that heterogeneity of individual protein synthesis rates caused the subset of proteins contributing to the apparent synthesis rates to shift over time. We further showed that not all proteins reach complete renewal indicating that the assumption that all proteins eventually reach 100% new is not true. The fact that some proteins do not reach complete renewal and do so at different rates can invalidate the assumption that the rate of tracer incorporation will maintain a linear phase. Finally, we demonstrated that the functional heterogeneity and synthesis rate differences across individual proteins within a bulk measure change the protein representation in a measured value. This change in protein representation results in comparisons that do not reflect ‘like to like’ when sampling at different periods during a labelling period. Our findings illustrate that proper study design for long‐term labelling studies is critical for understanding the underlying biology of protein synthesis.

By using a labelling time course, we showed that individual proteins reach plateau enrichment at different times. A plateau at less than 100% can happen two ways: There is a fraction of the protein pool that is not renewing, or the precursor enrichment is overestimated so that the product enrichment never reaches the precursor enrichment [21, 23, 26]. Because each of our individual proteins has a different plateau value, and some of the individual proteins reach 100% new, we can safely rule out that the precursor enrichment is systematically overestimated. Therefore, the evidence supports that this is a real biological phenomenon, which we have previously discussed the implications of [21, 26]. In short‐term amino acid labelling experiments on the scale of minutes to hours, label saturation is not a concern because measurements are made within the linear region of the labelling trajectory [8, 9]. However, in longer term labelling studies, commonly done with D_2_O, rapidly renewing proteins can reach a plateau value before the time of tissue sampling. With increasing time from the start of labelling to sampling, a greater number of proteins reach the plateau (demonstrated in Figure 4). At the plateau value, the individual protein stops contributing to an increase in fraction new, thus diminishing its contribution to the overall fraction new beyond this timepoint. Therefore, if one biopsy is used to establish protein synthesis rates over one period (e.g., baseline) and then a second one is taken after a second period (e.g., intervention), the number of individual proteins that have reached a plateau is greater in the second biopsy. To demonstrate this effect, we used the label accumulation from Day 0 to Day 4 as a baseline ‘pre‐intervention’ FSR and then explored the effect of increasing labelling periods on the calculated ‘intervention’ FSR. Without any treatment, the apparent synthesis rate decreased as the time window used to determine the ‘intervention’ FSR increased (Figure 3). Consequently, the FSR appears to decrease, despite the actual synthesis rate being unchanged.

A second consideration for interpreting the results of long‐term D_2_O labelling studies is how individual proteins, with individual rates of synthesis, contribute to bulk FSR over different labelling periods. Figure 4 shows individual fraction new traces of each protein over our labelling period. Note the heterogeneity in plateau value as discussed above but also the heterogeneity of synthesis rates. A consequence of this heterogeneity is that synthesis estimates from biopsies taken at different labelling timepoints reflect different protein contributions, because individual proteins label and plateau at different rates. The differences in rate and contribution to overall FSR are visualized in Figure 5B. As labelling time increases the rapidly synthesized proteins have a sharp early decline in contribution to FSR while the slowly synthesized proteins have a steady increase. It is helpful to consider the proteins that contribute to the FSR from Days 0–15 and Days 15–45, not as distinct time points, but as a continuum. A bulk synthesis rate created from Days 0–15 is driven by a substantially different set of proteins than the bulk synthesis rate from Days 15–45 as shown by the shift in the contribution of each synthesis decile in Figure 5B. Note how the slowest deciles grow dramatically in contribution, while the fastest deciles effectively disappear. It is common to subtract a baseline enrichment with serial biopsies to capture the labelling that is new in the subsequent periods. However, this approach assumes that the bulk enrichment signal accumulates linearly over time and reflects a consistent pool of proteins across labelling windows. Using LC–MS peptide‐level fraction‐new measurements, we show that apparent FSR varies with labelling duration as proteins approach plateau and that the proteins contributing to the measured synthesis shift over time. Comparing early and late ‘rates’ treats the proteome as if it were composed of a single homogeneous ‘uniprotein’, rather than a complex heterogeneous mixture of thousands of proteins.

The considerations noted also impact biological interpretation of the results. To illustrate the practical implications, consider the following representative experimental design: D_2_O consumption begins on day 0 and continues for a total of 15 days. In this example, there is a baseline phase lasting 5 days (ending on Day 4) and an intervention phase lasting 11 days (ending on Day 15). The differences in the contribution of each protein synthesis group to proteome‐wide synthesis are highlighted in our data with Figure 3B. The fast and slow groups contributed approximately equally (by design) at Day 4 (~20%), while at the 15‐day time point, the contribution of the fast synthesis group dropped to 12%, and the contribution of slow‐synthesis proteins rose to 23.7%. Consequently, proteins with lower plateau values and faster synthesis rates will contribute less to the observed FSR of the second biopsy compared to proteins with slower synthesis rates. This difference in contribution affects not only observed FSR but also the composition of the proteome contributing to FSR. When we examined the GO cellular component composition of the detected proteome and synthesis groups in our data (Figure 6) at different labelling periods, we observed a shift in the cellular component composition of proteins driving FSR. For example, in Figure 6B, myofibrillar/contractile proteins contributed substantially to the slow and average groups but minimally to the fast group. Consequently, at the 4‐day timepoint, these cytoskeletal and structural proteins made a disproportionately large contribution to the measured synthesis rate compared to the 15‐day time point. Therefore, differences between early and late ‘bulk’ synthesis measurements reflect a changing mixture of compartment classes rather than true treatment effects. Additionally, the subtraction of T0 (after Day 4) enrichment, commonly used in multiple‐biopsy experimental designs, does not account for differences in the relative contributions of protein groups (e.g., cytoskeletal proteins at early timepoints) to the bulk enrichment signal across cellular components and may therefore alter data interpretation. Although the specific boundaries used to define slow, average and fast synthesis groups were selected for analytical purposes, these groupings capture meaningful differences in protein behaviour that should be considered when designing long‐term D_2_O labelling studies. Further investigation into when distinct functional protein classes drive measured synthesis could inform the design of labelling schemes aimed at quantifying synthesis within specific protein populations.

One potential limitation of the current study is that we used mouse skeletal muscle, not human skeletal muscle. We performed the study in mice to avoid the number of muscle biopsies in human subjects that we would have needed to get the time resolution we desired. The exact rates of synthesis of the individual proteins and their summations might be different between mouse and human. However, the principle of the study and the interpretations remain unchanged whether in mouse or human skeletal muscle. There is nothing fundamentally different about the behaviour of mouse and human protein pools that would preclude the current findings being applicable to measurements of human muscle protein FSR. In addition, even though the current study was performed on skeletal muscle, the same principles would apply to other tissue protein pools.

In conclusion, as tracer‐based measurement of protein synthesis becomes increasingly accessible, we must revisit common assumptions inherited from short‐term labelling paradigms. Here, we show that in continuous long‐term labelling studies, biopsy timing alters both measured bulk and myofibrillar FSR and the relative contributions of individual proteins to that measurement. We also demonstrate that many proteins do not fully renew and that individual proteins are extremely heterogeneous in both renewal rate and renewal plateau. When taken together, these findings indicate that serial biopsies collected across separate labelling intervals cannot be interpreted as comparable measurements. We hope readers take away the importance of acknowledging the composition of measured synthesis and designing their future labelling studies with this in mind. The field therefore should use parallel group designs or initiate creative new approaches to circumvent these issues.

## Funding

This work was funded by Veterans Affairs (IK6BX007150) and National Institutes of Health (P30AG050911, 5F32AG087622, R00AG051735 and R01GM149762).

## Conflicts of Interest

The authors declare no conflicts of interest.

## Supporting information


**Table S1:** Proteins identified in a myofibrillar protein fraction using the fractionation procedure used in our previous publications for Supplementary Figure S1. Proteins that were matched to the proteome‐wide data set and used to calculate the myofibrillar FSR are indicated.

**Figure S1:** Influence of the different time‐periods of measurement on apparent FSRs of the myofibrillar (Myo) protein fraction and bulk protein fraction.

**Figure S2:** (A) Distribution of protein synthesis rates (half‐life) by slow (blue), average (green) and fast (red). (B) Distribution of protein asymptotes (plateau) by synthesis group.

**Figure S3:** (A) Protein abundance (log‐scaled) vs. protein rate constant (k, log scale). Colours indicate synthesis groups (B) Protein rate constant (k, log scale) vs. plateau fraction new value.

**Figure S4:** (A) Protein count per synthesis group. Colours indicate synthesis groups (B). The proportion of the total proteome mass that each synthesis group comprises.

**Data S1:** Supplementary material.

## Data Availability

The data that support the findings of this study are available from the corresponding author upon reasonable request.
