# Supplementary material for: Deuterium‐Oxide Labelling Proteomics Illustrates the Importance of Sample Timing and the Limitations of Serial Biopsies
Source: J Cachexia Sarcopenia Muscle. 2026 Nov 10;17(6):e70426. doi: 10.1002/jcsm.70426 (PMC13749624; doi:10.1002/jcsm.70426)
Supplement: Supplementary file 1 [file JCSM-17-e70426-s005.docx]

**Supplementary Methods for Deuterium-oxide labeling proteomics illustrates the importance of sample timing and the limitations of serial biopsies**

^‡^Colleen L. O’Reilly^1^, ^‡^Michael E. Taylor^1,2^, Kamil A. Kobak^1^, Ann Chiao^1^, Rovshan G. Sadygov^4^, Michael T. Kinter^1^, Benjamin F. Miller^1,3^

^1^Aging and Metabolism Research Program, Oklahoma Medical Research Foundation, Oklahoma City, OK, 73104, USA

^2^Department of Biochemistry and Physiology, University of Oklahoma Health Campus, Oklahoma City, OK, 73104

^3^Veterans Affairs Medical Center, Oklahoma City, OK, 73104, USA

^4^Department of Biochemistry and Molecular Biology, The University of Texas Medical Branch, Galveston, Texas 77555, USA

*Corresponding Author: Benjamin F. Miller, Aging and Metabolism Research Program, Oklahoma Medical Research Foundation (OMRF), Oklahoma City, OK, 73104. Phone: 405-271-7767. Email: [Benjamin-miller@omrf.org](mailto:Benjamin-miller@omrf.org)

^‡^These authors contributed equally to this work.

Keywords: Deuterium Oxide, skeletal muscle, stable isotopes, biopsy, proteomics

**Methods:**

*Animals and Ethical Approval:*

To avoid the logistical and practical constraints associated with multiple human biopsies, we used mouse skeletal muscle to test our hypothesis. All animal procedures were conducted in accordance with institutional guidelines for the care and use of laboratory animals and were approved by the Institutional Animal Care and Use Committee at the Oklahoma Medical Research Foundation (OMRF). Out of convenience and to make good use of rodent resources, we used mice that were undergoing a timecourse of labeling for other purposes (control mice from (Kobak *et al.*, 2025)). These mice were 23-month-old male C57Bl/6 mice that were housed in the vivarium at OMRF. Animals were group housed (a maximum of five per cage) with *ad libitum* access to food and water in a room on 14:10 light:dark cycle with constant temperature and humidity. Prior to sacrifice, mice were subjected to 4-hour fasting. For tissue and blood collection, mice were anesthetized with isoflurane followed by exsanguination through cardiac puncture for euthanasia.

*Experimental Design:*

To compare how the period of labeling impacts the contribution of individual proteins to an integrated protein synthesis rate, we used labeling periods of 4, 15, 30 and 45 days (n=3 mice/labeling period). At the onset of the labeling period, mice were given an intraperitoneal bolus injection of filtered 99% D_2_O (Sigma Aldrich. St. Louis, MO) equivalent to 5% of body water, estimated at 60% of body mass. The bolus injection was followed by 8% D_2_O-enriched drinking water for the remainder of the labeling period. At the end of the labeling period animals were euthanized when blood from a cardiac puncture was collected into EDTA-lined tubes. Blood was centrifuged at 3000 x *g* for 5 min. at room temperature. Serum was aliquoted and frozen at −80°C until further analysis. The gastrocnemius was subsequently removed, weighed, flash frozen and stored at -80°C. Muscle from all the mice were then processed as one batch for LC-MS/MS analysis **(Figure 1).**

*Body Water Enrichment:*

We determined body water enrichment as previously described (Bubak *et al.*, 2024). Briefly, 50 µL of plasma and 0%–12% D_2_O standards (Sigma, 151882) were placed into the inner well of an O-Ring screw cap and inverted on a heating block overnight at 80°C. Samples were diluted 1:300 in ddH_2_O and analyzed on a liquid water isotope analyzer (Los Gatos Research, Los Gatos, CA, USA) against a standard curve prepared with samples having different concentrations of D_2_O (0%–12%).

*Sample Processing:*

Frozen powdered skeletal muscle (~30 mg) was lysed in 1 mL RIPA buffer and sonicated for 15 cycles of 30 sec on/off (Bioruptor Plus; Diagenode, Denville, NJ). Samples were incubated at 90°C and 800 rpm for 10 min, and then the sonication was repeated. Then samples were centrifuged for 5 min at 500 *x* *g* to remove debris. The supernatant was collected and the protein concentration in sample was measured using Pierce 660 Protein Assay. 80 μg of total protein was taken for analysis, and 1 μg of BSA was added to the protein samples as an internal standard. Protein samples were desalted by precipitation in 1 mL of acetone overnight at −20°C. After acetone precipitation, we centrifuged the protein samples at 10,000 *x* *g* for 10 min and decanted the acetone. We then dried the samples via vacuum centrifugation (Thermo Scientific SpeedVac) at 45°C until a dry protein pellet formed. The protein pellet was solubilized in 80 μL Laemmli sample buffer (BioRad), and 20 μg protein was run in a 12% SDS-Page gel (BioRad Criterion system). The gels were fixed and stained with GelCode Blue Stain (Pierce Chemical Company). Each sample was cut from the gel as the entire lane and divided into smaller pieces. The gel pieces were washed to remove the Coomassie blue and then reduced in 50mM DTT, alkylated in 15mM iodoacetamide, and digested overnight with 1 μg trypsin per sample in 200 μL 50 mM ammonium bicarbonate. The mixture of peptides was extracted from the gel, evaporated to dryness in a SpeedVac, and reconstituted in 200 μL 1% acetic acid (v/v) for liquid chromatography (LC)-tandem mass spectrometry (MS) analysis.

*LC-MS/MS Analysis:*

All samples were analyzed on a ThermoScientific Orbitrap Exploris 480 interfaced to a Vanquish Neo HPLC, an EasySpray Source, and an EasySpray C18 PepMap Neo column. 10 µL sample volumes were injected and eluted from the column at 125 nL/min with a linear gradient of 2% CH_3_CN in water with 0.1% formic acid to 40% CH_3_CN in 50 min. The instrument was operated in data-dependent acquisition (DIA) mode, acquiring a full scan mass spectra with a resolution of 30,000 and subsequent MS/MS with an m/z resolution of 15,000.

LC-MS/MS files were analyzed using DIA-NN 2.2.0 (Demichev *et al.*, 2020; Demichev, 2026), following the instructions provided in the official documentation. Parameters used were: 1 missed cleavage, up to 2 variable modifications, cysteine carbamidomethylation as a fixed modification and methionine oxidation as a variable modification. Database searching was performed against the *Mus musculus* UniProtKB reference proteome (The UniProt Consortium, 2025). Following DIA-NN analysis, the main report was filtered for peak quality and retention time (specifics are supplied in the **Supplementary Materials**). The filtered main report was then imported into Skyline, where peaks were integrated and peak areas were exported. Analysis was completed on 2826 peptides across 308 proteins.

*Protein Synthesis Rate Calculation:*

The calculation of protein synthesis rates and asymptotes is shown in full in the **Supplementary Materials**. The following is a brief description of the workflow for the interested reader. For this experiment, we restricted our calculations to the M+0 and M+1 mass isotopologues, as these are typically the most abundant species and thus have the most reproducible spectra. These calculations were done for each peptide, and protein synthesis rates calculated using all peptides that unambiguously originate from that protein.

First, the relative isotope abundances (RIA) of the M+0 and M+1 mass isotopologues in M+0 and M+1 space were determined with the following equations. $M_{0}$ refers to the M+0 mass isotopologue, and $M_{1}$ refers to the M+1 mass isotopologue.

$$RIA M_{0}=\frac{M_{0}}{M_{0}+M_{1}} RIA M_{1}=\frac{M_{1}}{M_{0}+M_{1}}$$

Next, with known body water deuterium enrichment (BWE), known number of exchangeable hydrogens (NEH) per amino acid (Commerford *et al.*, 1983), and known peptide sequence, the theoretical *RIA* $M_{0}$ at complete labeling can be calculated. Since heavier (M+2, M+3, etc.) mass isotopologues have non-trivial abundances and we have restricted our calculations to M+0 and M+1, we must explicitly outline the relationship between the M+0 and M+1 mass isotopologues during labeling. The following equation was used:

$$Theoretical Minimum {RIA M}_{0}=$$

$$\frac{{RIA M}_{0} *\left( 1-BWE \right)^{NEH}}{{RIA M}_{0}*\left( 1-BWE \right)^{NEH}+RIA M_{1}*\left( 1-BWE \right)^{NEH}+{RIA M}_{0}\left( NEH \right)\left( BWE \right)\left( 1-BWE \right)^{NEH-1}}$$

The numerator is the unlabeled *RIA* $M_{0}$multiplied by the probability of incorporating zero deuterium labels, thereby representing the fraction of the peptide that is expected to remain M+0 at complete labeling. In the denominator, the first term is the unlabeled *RIA* $M_{0}$multiplied by the probability of incorporating zero deuterium labels (described in the preceding sentence). The second term is the unlabeled *RIA* $M_{1}$ (peptides containing one naturally occurring heavy isotope, such as ¹³C or ¹⁵N) multiplied by the probability of incorporating zero deuterium labels (remaining at M+1), which is the fraction of unlabeled M+1 peptide that is expected to remain M+1 at complete labeling. The third term is the unlabeled *RIA* $M_{0}$multiplied by the probability of incorporating exactly one deuterium label (shifting to M+1), which is the fraction of M+0 that is expected to become M+1 at complete labeling. The three denominator terms exhaustively describe every pathway by which a new peptide can appear as M+0 or M+1.

Then, using the *RIA* $M_{0}$ from an unlabeled sample as a baseline, the fraction of a peptide that was newly synthesized by each timepoint was calculated by comparing the *RIA* $M_{0}$ from an experimental sample (*ex.* 4 days, 15 days, 30 days of labeling) to the theoretical minimum *RIA* $M_{0}$ at complete labeling:

$$Fraction New=\frac{{Unlabeled RIA M}_{0}- Experimental {RIA M}_{0}}{Unlabeled {RIA M}_{0}-Theoretical Minimum {RIA M}_{0}}$$

Once the fraction new of each peptide was determined at each timepoint, the protein fraction new value was calculated as the mean of the peptide fraction new values. Peptides were filtered for quality prior to being included in the mean (see **Supplementary Materials**). Then, by fitting the following equation to the fraction new value of a protein at each timepoint, *k* (rate) and *A* (asymptote) values were determined:

$$Fraction New=A*(1-e^{-kt})$$

*Bulk FSR Estimation:*

To re-capitulate the total-muscle (i.e., bulk) FSR measurements commonly reported from GC-MS analyses of hydrolyzed whole muscle, we determined the fraction new of each protein at each timepoint and created an abundance-weighted sum of these values to calculate a proteome-wide fraction new at each timepoint. The FSR at each timepoint was calculated by dividing the fraction new by the length of the labeling period.

*Myofibrillar FSR Estimation*

To re-capitulate the myofibrillar FSR measurements commonly reported from GC-MS analyses of hydrolyzed myofibrillar fractions, we performed a myofibrillar fractionation on a mouse muscle aliquot. The sample was analyzed via LC-MS/MS to determine what proteins were present in the myofibrillar fraction. For each matched protein, fraction new was estimated at each timepoint using its fitted turnover parameters from the proteome-wide dataset. These values were weighted by the corresponding protein abundances in the proteome-wide dataset, normalized to sum to one across the matched proteins included in the analysis. The weighted values were then summed to calculate a pooled myofibrillar fraction new at each timepoint. The FSR (%/day) at each timepoint was calculated by dividing the fraction new by the length of the labeling period.

*Gene Ontology Enrichment Analysis*:

After LC-MS/MS analysis all proteins found to meet all quality control requirements were included in our Gene Ontology analysis (GO). The list of proteins was analyzed using GO enrichment analysis for cellular component. All cellular components listed were then distilled into 7 categories (cytoskeleton/structural, Extracellular Matrix/Junctions, Membranes & Transport, Mitochondria & Oxidative Metabolism, Myofibrillar/Contractile, Nuclear, and Sarcoplasmic Reticulum/Calcium). Proteins could appear in multiple categories. To determine the cellular component makeup of the proteome, as defined by GO, we calculated the abundance-weighted proteins per category. Each protein contributes its full abundance to each category it belongs to but was only counted once within a category. Each category’s contribution to the total proteome is calculated using the below equation. This approach allows the contribution of each protein to reflect its relative abundance in the proteome while still permitting proteins with multiple annotations to contribute to all relevant GO categories. We determined this would provide the most physiologically representative view of proteome composition.

$Category A\%= \frac{\sum abundance of each protein counted within category a}{\sum abundance of all protein-category counts across all categories}$

To account for the influence of protein synthesis kinetics on functional representation at a given sampling time, we quantified GO category composition separately within each synthesis group (fast, average, slow). For each group, we calculated the fraction of total protein abundance attributable to each GO category. This approach allowed us to determine how functional representation differs as a function of synthesis rate, thereby illustrating how proteins contributing to GO categories would vary across sampling intervals.

$\%turnover group that is category A= \frac{\sum abundance of each protein in category A (within turnover group)}{\sum abundance of all proteins (within turnover group)}$
